# *Schistosoma japonicum* extracellular vesicle proteins serve as effective biomarkers for diagnosing parasite infection

**DOI:** 10.3389/fcimb.2024.1391168

**Published:** 2024-05-16

**Authors:** Huixin Wu, Bikash R. Giri, Huimin Li, Yameng Zheng, Xiaoli Yan, Guofeng Cheng

**Affiliations:** ^1^ Shanghai Tenth People’s Hospital, Tongji University School of Medicine, Shanghai, China; ^2^ Department of Zoology, Kuntala Kumari Sabat (KKS) Women’s College, Balasore, Odisha, India; ^3^ Shanghai Veterinary Research Institute, Chinese Academy of Agricultural Sciences, Key Laboratory of Animal Parasitology of Ministry of Agriculture, Shanghai, China

**Keywords:** schistosomiasis, *Schistosoma japonicum*, recombinant protein, indirect ELISA, diagnosis

## Abstract

*Schistosoma* species are the causative agent of schistosomiasis and shows worldwide distribution. There is a great need to develop a sensitive diagnostic approach for controlling the disease. Previously, we identified large numbers of Extracellular Vesicle (EV) proteins from *Schistosoma japonicum* (*S. japonicum*), but rarely these proteins have been evaluated for their diagnostic potential. In the present study, we performed bioinformatic analyses of *S. japonicum* identified EV-associated proteins from the previous study and then identified *Schistosoma*-specific proteins with potentially secreted capability. Among them, we selected SJCHGC02838 protein, SJCHGC05593 protein, SJCHGC05668 protein and a hypothetical protein (SJHYP) to evaluate their diagnostic potential for detecting *S. japonicum* infection. First, we determined the expression of these four proteins at the transcript levels using qRT-PCR and revealed that all these genes showed higher expression in adult stage. Then, we cloned the full-length cDNA for each protein into a prokaryotic expression vector and successfully generated the recombinant proteins. Upon the purification of recombinant proteins, we developed an indirect ELISA method to evaluate the diagnostic potential of these purified recombinant proteins. The results showed high sensitivity for detecting *Schistosoma* infection. Additionally, these proteins also displayed a good potential for detecting *Schistosoma* infection, especially SJCHGC05668 protein at an early stage. The diagnostic potentials of these recombinant proteins were further evaluated by Western blot and comparatively analyzed by our previously developed cfDNA methods.

## Introduction

Schistosomiasis is a neglected tropical disease caused by *Schistosoma* species, particularly *S. japonicum*, *S. mansoni* and *S. haematobium* (Colley et al., 2014) and about 251.4 million people require preventive treatment for schistosomiasis (Kokaliaris et al., 2022). *S. japonicum* is mainly prevalent in China, Philippines, and Indonesia (Chitsulo et al., 2000). Lacking a sensitive diagnostic tool is the main obstacle to eliminate this disease.

The traditional diagnostic method is to check the eggs in the stool or urine samples by microscopy. The method is time-consuming and lacks sensitivity, particularly for low-intensity infection (Rabello et al., 2002). Recent studies have focused on immunodiagnosis of schistosomiasis since it is more sensitive and less time-consuming than the traditional method. Although several diagnostic antigens such as soluble egg antigen (SEA), soluble worm antigens (SWA) and excretory and secretory (ES) products were shown to have good potential for diagnosing schistosomiasis, cross-reactions with antibodies from other parasite infections were observed to some extent (Zhou et al., 2008; Ludolf et al., 2014; Sotillo et al., 2016). Hence, researchers have focused on pure *Schistosoma*-specific proteins for serological diagnosis. To date, several diagnostic biomarkers have been identified and characterized. For example, the members of the saposin-like proteins (*Sj*SALP) family were highlighted for schistosomiasis diagnosis. *S. mansoni*-SLP-1 (a member of *Sm*SALP family) was shown to be specifically recognized by serum samples from *S. mansoni*-infected mice and patients (Don et al., 2008). Furthermore, *Sj*SP-13, another member of *Sj*SALP, was also evaluated as a diagnostic antigen and a 6-fold increase in sensitivity has been achieved (Xu et al., 2014). In addition, *Sj*SALP4 and *Sj*SALP5, from *Sj*SALP family, could also be recognized by serum samples from both *S. japonicum*-infected laboratory animals and patients, with a higher sensitivity of 98% and 96%, respectively, and 100% specificity (Liu et al., 2016). In addition to members of SALP family, *S. japonicum* proteins such as cathepsin B (Macalanda et al., 2019), thioredoxin peroxidase-1 (Angeles et al., 2011, Angeles et al., 2012; Macalanda et al., 2018) and multidrug resistance-associated protein 1 (Feng et al., 2017) have shown good diagnostic capability for detecting *S. japonicum* infection.

*S. japonicum* extracellular vesicles (*Sj*EVs) have been found to play important roles in parasite-host interactions. It was shown that *Sj*EVs could be implicated in the pathogenesis of schistosomiasis via transferring their cargo miRNA to hosts (Zhu et al., 2016) and it can mediate M1-type immune-activity of macrophage (Wang et al., 2015). More recently, *Sj*EVs were shown to be uptake by macrophages and peripheral blood immune cells and their miRNA cargo can be transferred to recipient cells, resulting in increased macrophage proliferation and TNF-α production (Liu et al., 2019). In addition to functional miRNA, numerous proteins were identified from the secreted EVs (Zhu et al., 2016), suggesting that these proteins may be easily recognized by host immune system, which could serve as effective biomarkers for diagnosing parasite infection. A recent report found that CD63 identified from *Sj*EVs exhibited diagnostic potential (Wang et al., 2018) and another study predicted the epitope of several *Sj*EV proteins and found two of the antigens having diagnostic potential (Chen et al., 2020). Further combining these two EV protein epitopes demonstrated that *Sj*EV proteins could serve as potential diagnostic markers (Chen et al., 2020).

In the present study, we performed bioinformatic analyses based on our previously identified EV associated proteins from *S. japonicum* adults and then evaluated the diagnostical potential of *Schistosoma*-specific proteins with potentially secreted capability. The results indicated that these selected proteins displayed high sensitivity for detecting *Schistosoma* infection, particularly SJCHGC05668 protein at an early stage. Their diagnostic potentials of these recombinant proteins were further confirmed by Western blot and ELISA and were also comparatively analyzed by our previously developed cfDNA methods.

## Materials and methods

### Ethics approval for animal experiments and collection of parasite materials

All animal experiments were carried out in strict accordance with the recommendations in the Guide for the Care and Use of Laboratory Animals of the Ministry of Science and Technology of the People’s Republic of China. All efforts were made to minimize suffering. All animal procedures were approved by the Animal Management Committee and Technology Commission of Shanghai municipal government for Shanghai Veterinary Research Institute, Chinese Academy of Agricultural Sciences, China (Permit number: SYXK 2016–0010). The life cycle of *S. japonicum* (Jiangxi isolate) was maintained in Kunming mice (Shanghai JieSiJie Laboratory Animal Co., Ltd, Shanghai, China) and *Oncomelania hupensis* was obtained from Center of National Institute of Parasitic Disease, Chinese Center for Disease Control and Prevention, Shanghai, China. Mice were infected with approximately 200 cercariae, respectively via the skin of the abdomen. Before infection and at 5-, 10-, 22- and 28-days post-infection (dpi), blood was collected from the mice by retro-orbital bleeding. After collection, the whole blood was allowed to clot at room temperature for 30 min, centrifuged at 3000 ×g for 10 min at 4°C. The resulting sera were collected and stored at -80°C until use. In addition, human serum samples infected with *S. japonicum* were provided by Institute of Biology, University of the Philippines Diliman. Worms were collected from infected mice by perfusion at 7, 14, 21 and 28 dpi. Eggs were isolated from the livers of infected animals as described elsewhere (Lewis et al., 1986). All collected parasites were stored in liquid nitrogen until further use.

### Analysis and selection of the four *S. japonicum* extracellular vesicle proteins

Based on our previous study, Zhu et al. (2016) identified 403 proteins in *S. japonicum* EVs by LC-MS/MS, and there were 116 proteins identified from a total of 9 gel blocks. SJCHGC Hypothetical protein (UniProtKB/SwissProt ID: C1L4B9), SJCHGC02838 protein (UniProtKB/SwissProt ID: Q5DGI7), SJCHGC05593 protein (UniProtKB/SwissProt ID: Q5BW52), and SJCHGC05668 protein (UniProtKB/SwissProt ID: Q5DD37) were selected because they all possess signal peptide and are predicted to be *Sj*EVs structure-associated and secreted proteins (Zhu et al., 2016), one of which previously showed diagnostic potential (Wang et al., 2018).

### Expression profiles of four *S. japonicum* EV proteins at transcript levels

Quantitative real-time polymerase chain reaction (qRT-PCR) was performed to analyze the expression profiles of these four *S. japonicum* genes in eggs, cercariae, and parasites collected at 7, 14, 21 and 28 dpi. Total RNA was isolated from eggs and parasites from different developmental stages using TRIzol reagent (Life Technologies, Carlsbad, CA, USA). The isolated RNA was quantified using a Nanodrop ND-1000 spectrophotometer (Nanodrop Technologies, Wilmington, DE, USA) and stored at -80°C until further use. To determine the transcriptional levels of these genes at different developmental stages of *S. japonicum*, 500 ng RNA from each stage was reverse transcribed to cDNAs using a PrimeScript RT reagent Kit (TaKaRa, China). The level of mRNA of these genes under different conditions were analyzed by qRT-PCR using specific primers as follows: the forward primer for SJHYP 5’: GTTAGTCTTTATGTTGGTTGTCCTT and the reverse primer for SJHYP 3’: CAATTCTACTTGATTACTTCTCGATT, the forward primer for SJCHGC05668 protein 5’: AAAAGTAAATCAGAAAGTGTGTATTG and the reverse primer for SJCHGC05668 protein 3’: AGTTCAAACAGGAATAACAACTGA, the forward primer for SJCHGC05593 protein 5’: CGATTAAATCTTTCGCAGACC and the reverse primer for SJCHGC05593 protein 3’: ATCTTCAAAGGGTATTGGATGT, the forward primer for SJCHGC02838 protein 5’: AGTGAGATCATCAGGATGGTTAG and the reverse primer for SJCHGC02838 protein 3’: AGAGGAGCCTGTCACAGATA. *S. japonicum* nicotinamide adenine dinucleotide dehydrogenase (NADH) gene was used as an internal control using the forward primer: CGAGGACCTAACAGCAGAGG and the reverse primer: TCCGAACGAACTTTGAATCC (Liu et al., 2012). qRT-PCR was performed using SYBR Premix Ex Taq (TaKaRa) using the following cycling conditions: 95°C for 30 s, followed by 40 cycles of amplification (95°C for 5 s, 60°C for 30 s, 72°C for 20 s). Relative mRNA expression was calculated using the 2^−ΔCt^ method (Livak and Schmittgen, 2001).

### Cloning, expression and purification of recombinant proteins

The extracted total RNA of *S. japonicum* adult worms was reverse transcribed to cDNA as described above. The full-length of *SJHYP*, *SJCHGC02838*, *SJCHGC05593* and *SJCHGC05668* were amplified by PCR from the reverse transcribed cDNA using the forward primer for SJHYP-5’: GACAGCCCAGATCTGGGTACCATGAAACTTGTGTTAGTCTTTATG and the reverse primer for SJHYP-3’: GGTGCTCGAGTGCGGCCGCAAGCTTTCATTCCTCACGAGTAGAC, the forward primer for *SJCHGC02838–*5’: GACAGCCCAGATCTGGGTACCATGTGGTCAATATTCATCTTG and the reverse primer for *SJCHGC02838–*3’: GGTGCTCGAGTGCGGCCGCAAGCTTTCACTCGATCGATTTTCTC, the forward primer for *SJCHGC05593–*5’: GACAGCCCAGATCTGGGTACCATGGGGGGCTTGTTTTCAG and the reverse primer for *SJCHGC05593–*3’: GGTGCTCGAGTGCGGCCGCAAGCTTTTATAATATCTTGAAGTGCAGATTTATTTTTTCAATTTTC, the forward primer for *SJCHGC05668–*5’: GACAGCCCAGATCTGGGTACCATGAACAGTGGTTTTAAATTC and the reverse primer for *SJCHGC05668–*3’: GGTGCTCGAGTGCGGCCGCAAGCTTTTATAGAATGTATCCACTGATTG. The amplification reaction contained 12.5 μL 2×Hieff^®^PCR Master Mix (With Dye) (Yeason, Shanghai, China), 2 μL cDNA, 0.4 μM primers, and ddH_2_O up to 25 μL. The PCR conditions were, 94°C for 5 min followed by 35 cycles of 94°C for 30 s, 59°C for 30 s, 72°C for 90 s and a final extension step of 72°C for 10 min. Then purified amplicons were cloned into plasmid pET32a (+) using double-restriction digestion of *Kpn*I and *Hind*III. The recombinant plasmids were transformed into *Escherichia coli* DH5α (Novagen, Germany) and further confirmed by double restriction digestion and sequencing (Shanghai Jieyi Biotechnology Co., Ltd, Shanghai, China).

The recombinant plasmids with correct insertion were transformed into *E. coli* BL21(DE3) (Novagen) competent cells for the expression of these four recombinant proteins. The fusion proteins with His-tags were induced by adding 0.5 mM isopropyl β-d-1-thiogalactopyranoside (IPTG) at 37°C and 220 rpm. After 8 h, bacterial cells were collected and broken by sonication for 30 min on ice at 10 s intervals (Sonics & Materials Inc., Newtown, CT, USA). The enriched recombinant proteins were purified using the Ni-NTA Agarose (TYHF Biological Science and Technology Co., Ltd, Wuhan, China) according to the manufacturer’s instructions. To check the purification outcome, the recombinant proteins were separated using 12% sodium dodecyl sulfate-polyacrylamide gel electrophoresis (SDS-PAGE) under reducing conditions and stained in Coomassie Brilliant Blue G-250. The photos were taken by iBright image system (ThermoFisher Scientific, USA). The concentrations were determined by a BCA protein assay kit (Beyotime, Shanghai, China) and purified proteins were aliquot and stored at -80°C until further use.

### Western blot

The purified recombinant proteins were separated using 12% SDS-PAGE under reducing conditions, transferred onto polyvinylidene difluoride (PVDF) membranes (Bio-Rad, Hercules, CA, USA), and blocked in phosphate-buffered saline (PBS, pH 7.4) containing 0.1% Tween-20 (PBST, Sigma-Aldrich, St. Louis, MO, USA) and 5% non-fat dry milk for 2 h at room temperature. The membranes were incubated overnight at 4°C with *S. japonicum*-infected mice sera (1:200 dilution) collected at 7, 14, 21 and 28 dpi separately and washed 3 times for 10 min each with PBST. The membranes were further incubated with the secondary goat anti-mouse immunoglobulin (Ig) G antibody conjugated with horseradish peroxidase (1:5000 dilution; BWBIO, Beijing, China) at room temperature for 1 h. After three washes with PBST, the membrane was developed using the Immobilon Western kit (Millipore, Billerica, MA, USA). The immunoreactive bands were detected by iBright image system (ThermoFisher Scientific).

### Enzyme-linked immunosorbent assay conditions of four recombinant proteins recognized by mice sera

Several pre-experiments were conducted to determine the coating concentration of recombinant proteins (2 μg/mL), the dilution of mice serum (1:200), the optimal dilution of the secondary antibody (1: 5000), the optimal reaction time (15 min) of substrate solution. The 96-well ELISA plates (BBI, Sangon Biotech, Shanghai, China) coated with 100 µL/well of each protein diluted in carbonate buffer (disodium carbonate [Na_2_CO_3_]: 1.59 g/L and sodium bicarbonate [NaHCO_3_]: 2.93 g/L) were incubated overnight at 4°C. Non-specific binding was blocked using 200 µL/well PBS (pH 7.4) containing 0.1% Tween-20 (Sigma-Aldrich) and 5% non-fat dry milk for 1 h at 37°C. Then, the plates were washed 5 times for 2 min each with PBST, and incubated with 100 µL/well serum diluted with PBST for 1 h at 37°C. After five washes with PBST, 100 µL/well horseradish peroxidase-conjugated goat anti-mouse (to detect mice sera) or rabbit anti-human (to detect human sera) IgG (Beijing CoWin Biotech Co., Ltd. Beijing, China) diluted with PBST was added and incubated at 37°C for 1 h. The plates were washed five times with PBST, 100 µL/well 3,3′,5,5′-tetramethyl benzidine dihydrochloride (TMB) single-component substrate solution (Solarbio, Beijing, China) was added, and the reaction was stopped 15 min later using 50 µL/well ELISA Stop Solution (Solarbio). The absorbance was determined using a microplate reader (BioTek, USA) at 450 nm and each ELISA included a negative serum control.

ELISA was used to identify the levels of the four purified recombinant proteins separately recognized by serum of mice (n=5) infected with *S. japonicum* 200 cercariae collected at 5, 10, 22 and 28 dpi respectively, each ELISA included a negative serum control. Besides, based on the levels of four recombinant proteins and their mixtures recognized by the serum of mice (n=16) at 28 dpi, the diagnostic potentials of rSJHYP, rSJCHGC02838, rSJCHGC05593, rSJCHGC05668 separately and their equal mixture were preliminarily evaluated and compared. In addition, we also determined the diagnostic potential of these four proteins for mice infected with 10 and 20 cercariae at 3 and 5 dpi. The different number of *S. japonicum* cercariae for mice infection (10, 20, 200 cercariae) was also evaluated (n=3).

### ELISA conditions of SJCHGC05668 recombinant proteins recognized by human sera

ELISA conditions were optimized for different coating concentrations (2, 4, 6 μg/mL), serum dilutions (1:100, 1:200, 1:400 and 1:800), secondary antibody dilutions (1: 2500, 1: 5000, 1: 10000, 1: 20000), and the reaction time of substrate solution (5, 10, 15, 20 min). The rSJCHGC05668-indirect ELISA results of 38 human serum samples were compared with PCR results using cfDNA extracted from human sera as templates. The positive and negative sera were ratio diluted (1:100, 1:200, 1:400, 1:800,1:1600, 1:3200, 1:6400 and 1:12800) respectively to test the sensitivity of the human sera for this method. Five human serum samples were selected randomly to detect and evaluate the repeatability of intra and inter batch test results.

### Circulating cell-free DNA isolation

cfDNA was isolated from human serum samples using QIAamp Circulating Nucleic Acid kit following the manufacturer’s instructions. The isolated DNA was eluted using 25–50 µL of RNase-free water and measured the concentration and purity of eluted DNA were in a Nanodrop2000 spectrophotometer (Thermo Fisher Scientific).

### Statistical analysis

The data are represented as mean ± standard error (S.E.) derived from triplicate experiments. The significant differences between two groups were compared using the Student’s T-test considering *P* ≤ 0.05 is statically significant. The diagnostic potentials of the recombinant proteins were preliminarily evaluated by Receiver Operating Characteristic (ROC) analysis. We used GraphPad Prism 8.0 software to perform statistical analyses and plot the graphs and curves.

## Results

### Expression profiles of four *S. japonicum* EV proteins at transcript levels

**Figure 1:**
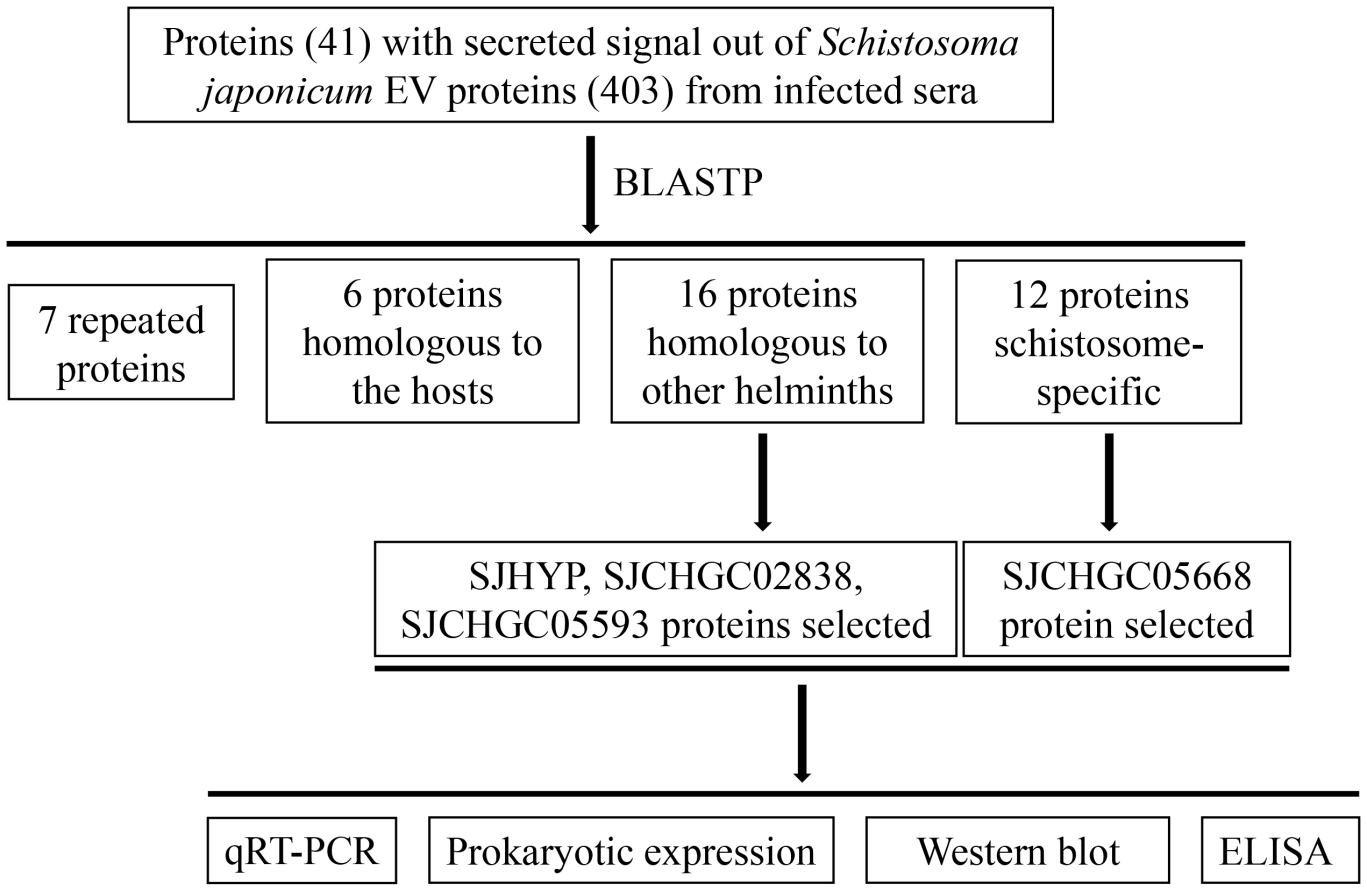
Flow-chart for analyzing and selecting the four *S. japonicum* extracellular vesicle proteins.

**Figure 2:**
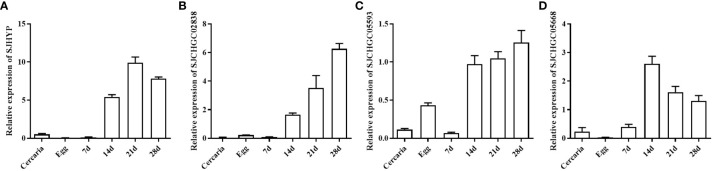
Expression profiles of four *S. japonicum* EV genes at different developmental stages of *S. japonicum*. Quantitative real-time PCR (qRT-PCR) was used to analyze the relative mRNA expression levels of these genes including SJHYP **(A)**, SJCHGC02838 **(B)**, SJCHGC05593 **(C)** and SJCHGC05668 **(D)** in eggs, cercariae, and parasites collected at 7, 14, 21 and 28 dpi. Relative mRNA expression levels were calculated using the 2^−ΔCt^ method. Representative results are shown as means and standard errors derived from triplicate experiments.

Based on our previous study (Zhu et al., 2016), we performed bioinformatics analysis and identified 41 proteins with secreted signal peptides out of 403 *S. japonicum* EVs proteins that could be recognized by the *S. japonicum* infected sera. Further BLASTP analysis revealed that out of 41 proteins segregated into 7 repeated proteins, 6 proteins were homologous to the host, 16 proteins were homologous to other helminths and 12 were schistosome-specific (**Figure 1**). Out of these proteins 16 were homologous to other helminths and 12 were schistosome specific proteins, we selected the proteins namely SJHYP, SJCHGC02838 and SJCHGC05593; and SJCHGC05668 protein, respectively because these proteins were showing highest similarity to *S. mansoni*. Expression profiles of the transcript level of these four genes in eggs, cercariae and parasites collected at 7, 14, 21 and 28 dpi were determined by qRT-PCR analysis. The results indicated that these four genes were expressed in all of the developmental stages investigated; showed higher expression in worms collected at 14, 21 and 28 dpi than in worms collected at 7 dpi and were expressed at relatively low levels in cercariae and eggs (**Figures 2A-D**). Results suggest that these four *S. japonicum* EV proteins are probably important in the regulation of adult development.

### Cloning, expression and purification of SJHYP, SJCHGC02838, SJCHGC05593, SJCHGC05668 recombinant proteins

**Figure 3:**
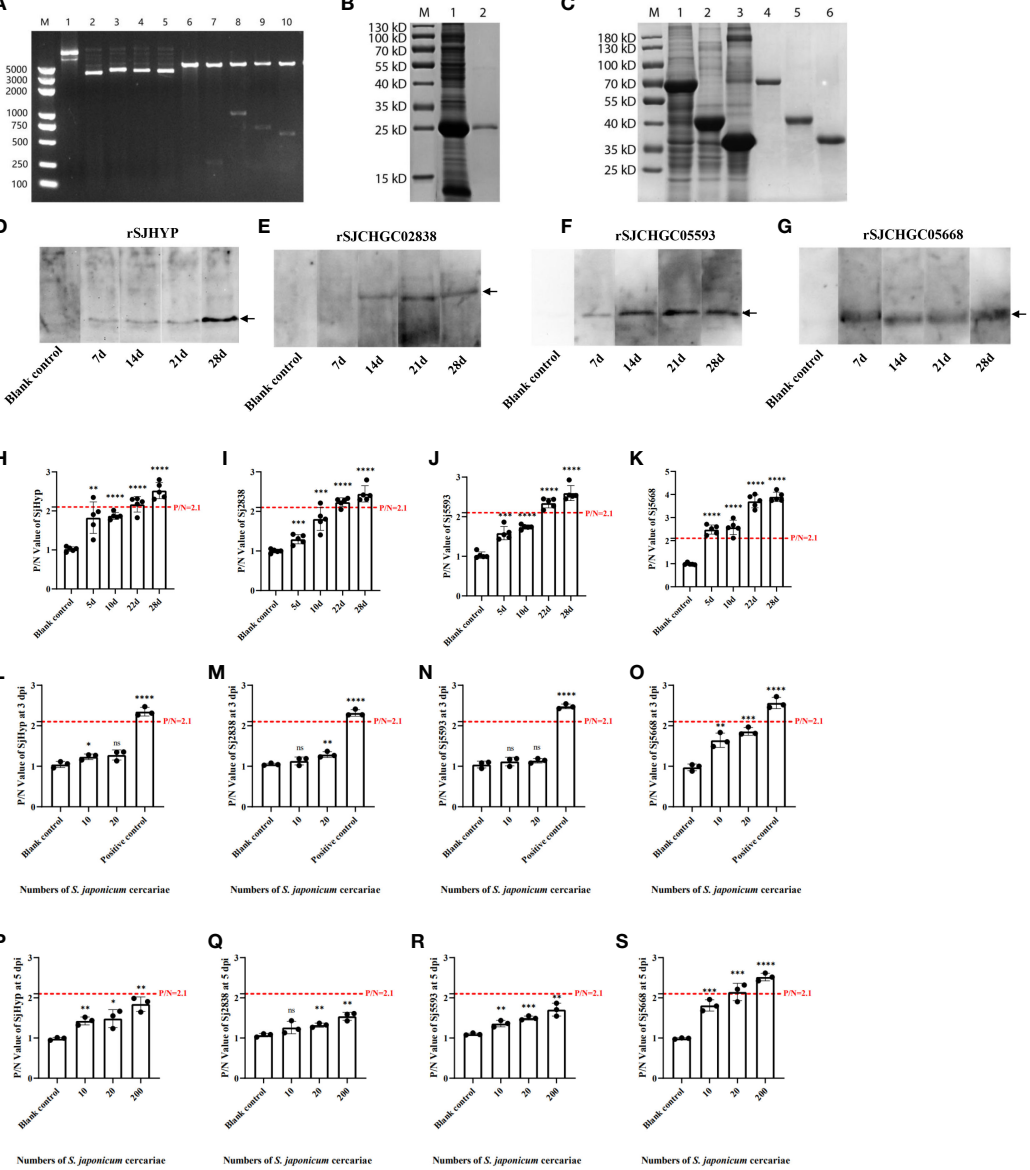
Cloning, expression, purification and immunological analyses of the SJHYP, rSJCHGC02838, SJCHGC05593 and SJCHGC05668 recombinant proteins recognized by sera from mice infected with *S. japonicum* cercariae. **(A)** Identification of four recombinant plasmids by double restriction digestion. M, marker; 1, 2, 3, 4 and 5 represents pET32a(+), pET32a(+)-SJHYP, pET32a(+)-SJCHGC02838, pET32a(+)-SJCHGC05593, and pET32a(+)-SJCHGC05668 recombinant plasmids, respectively; 6, 7, 8, 9 and 10 represents pET32a(+), pET32a(+)-SJHYP, pET32a(+)-SJCHGC02838, pET32a(+)-SJCHGC05593, pET32a(+)-SJCHGC05668 recombinant plasmids after restriction digestion, respectively. **(B)** Expression and purification of SJHYP recombinant protein. M, marker; 1, SJHYP recombinant protein induced by IPTG; 2, purified SJHYP recombinant protein. **(C)** Expression and purification of SJCHGC02838, SJCHGC05593 and SJCHGC05668 recombinant proteins. M, marker; 1, 2 and 3 represent SJCHGC02838, SJCHGC05593, SJCHGC05668 recombinant proteins induced by IPTG, respectively; 4, 5 and 6 represent purified rSJCHGC02838, rSJCHGC05593 and rSJCHGC05668 recombinant proteins, respectively. Western blot analysis of SJHYP **(D)**, rSJCHGC02838 **(E)**, rSJCHGC05593 **(F)** and rSJCHGC05668 **(G)** recognized by sera from mice infected with *S. japonicum* at different times of post-infection. Analysis of SJHYP **(H)**, rSJCHGC02838 **(I)**, rSJCHGC05593 **(J)** and rSJCHGC05668 **(K)** recombinant proteins recognized by sera from mice (n=5) infected with approximately 200 *S. japonicum* cercariae at different times of post-infection by Enzyme-linked immunosorbent assay (ELISA). Serum samples were collected from mice at 5, 10, 22 and 28 dpi. Analysis of SJHYP **(L)**, rSJCHGC02838 **(M)**, rSJCHGC05593 **(N)** and rSJCHGC05668 **(O)** recombinant proteins recognized by sera from mice (n=3) infected with approximately 20 *S. japonicum* cercariae at 3 by ELISA. Analysis of SJHYP **(P)**, rSJCHGC02838 **(Q)**, rSJCHGC05593 **(R)** and rSJCHGC05668 **(S)** recombinantproteins recognized by sera from mice (n=3) infected with approximately 10, 20 and 200 *S. japonicum* cercariae at 5 dpi by ELISA. Data are represented as means± S.E. and the significant differences between two groups were compared using Student’s T-test considering *p ≤ 0.05, **p ≤ 0.01, ***p ≤ 0.001, and ****p ≤ 0.0001 (Student’s t-test).

Complete sequences of Opening Reading Frames for SJHYP (261 bp), SJCHGC02838 (1524 bp), SJCHGC05593 (720 bp) and SJCHGC05668 (579 bp) were amplified by PCR using the cDNA reversely transcribed from the total RNA isolated from *S. japonicum* adult worms as templates and then cloned into plasmid pET-32a(+), respectively (**Figure 3A**). These four recombinant proteins were expressed in *E. coli* BL21(DE3) and purified using Ni-NTA agarose. SDS-PAGE analysis showed all four His-tagged recombinant proteins were purified and their rough molecular weight with 26 kDa (rSJHYP), 75 kDa (rSJCHGC02838), 44 kDa (rSJCHGC05593) and 37 kDa (rSJCHGC05668) corresponded to their expected sizes (**Figures 3B, C**).

### Immunological analysis of four recombinant proteins using sera collected from *S. japonicum*-infected mice

To evaluate immune responses against four *S. japonicum* EV proteins in final hosts, we used Western blot to test the immunological recognition of these recombinant proteins at different time points of post-infection. Serum samples were collected from mice before infection and at 7, 14, 21 and 28 dpi. The results showed that all the four recombinant proteins could be specifically recognized by the serum samples of *S. japonicum*-infected mice (**Figures 3D-G**). Specifically, rSJHYP could be recognized by serum samples collected at 7–21 dpi and stronger recognition was observed with serum samples at 28 dpi (**Figure 3D**); rSJCHGC02838 could be specifically recognized at 14, 21 and-28 dpi (**Figure 3E**); SJCHGC05593 could be weakly recognized by the serum samples collected at 7 dpi and the recognition is stronger with serum samples collected at 14, 21 and 28 dpi (**Figure 3F**); rSJCHGC05668 could be recognized by serum samples collected at 7, 14, 21 and 28 dpi (**Figure 3G**). Furthermore, ELISA was used to identify the levels of the four purified recombinant proteins separately recognized by serum of mice infected with *S. japonicum* cercariae collected at 5, 10, 22, and 28 dpi respectively (**Figures 3H-K**). P/N value greater than or equal to 2.1 is considered positive. Compared with the results of Western blot, it exhibited similar trends, all four recombinant proteins could be diagnosed as positive at 28 dpi and rSJCHGC05668 could be diagnosed as positive as early as 5 dpi. These results suggested that all four *S. japonicum* EV proteins can serve as potential diagnostic antigens but the diagnostic potentials varied. To further evaluate the potential of four *Sj*EV proteins as effective biomarkers for early diagnosis, we defined mice infected with approximately 10 and 20 cercariae as having a known level of infection. ELISA was used to identify the levels of the four purified recombinant proteins separately recognized by serum collected at 3 and 5 dpi respectively (**Figures 3L-S**). It was shown that only rSJCHGC05668 could be diagnosed as positive at 5 dpi, even at low levels of infection.

**Figure 4:**
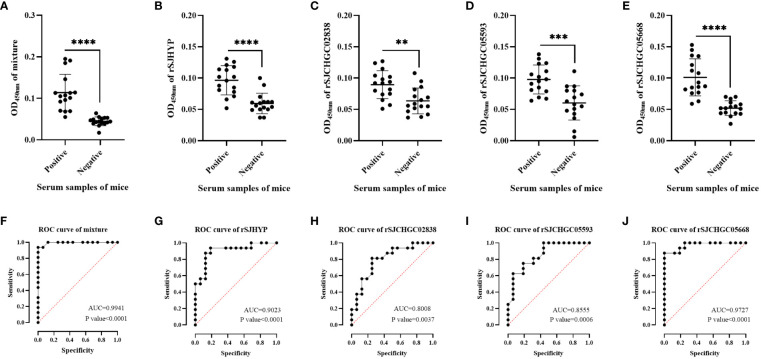
Analysis and comparison of the equal mixture of four recombinant proteins **(A)**, rSJHYP **(B)**, rSJCHGC02838 **(C)**, rSJCHGC05593 **(D)**, rSJCHGC05668 **(E)** respectively for the diagnosis of mice (n = 16) infected/uninfected with *S. japonicum* at 28 dpi using ELISA. The diagnostic potentials of the equal mixture of four recombinant proteins **(F)**, rSJHYP **(G)**, rSJCHGC02838 **(H)**, rSJCHGC05593 **(I)**, rSJCHGC05668 **(J)** were preliminarily evaluated by receiver operating characteristic (ROC) analysis. Data are represented as means ± S.E. and the significant differences between two groups were compared using Student’s T-test considering *P* ≤ 0.05 is statically significant. **p ≤ 0.01, ***p ≤ 0.001, and ****p ≤ 0.0001.

Therefore, we used ELISA to further identify the levels of four recombinant proteins and their mixtures recognized by the serum of mice at 28 dpi which was positive and before infection which was negative (**Figures 4A-E**). The diagnostic potentials of the rSJHYP, rSJCHGC02838, SJCHGC05593, rSJCHGC05668 and the equal mixture of four recombinant proteins were preliminarily evaluated by Receiver Operating Characteristic (ROC) analysis (**Figures 4F-J**). The Area under Curve (AUC) of the mixtures was highest but rSJCHGC05668 was pretty close. Besides, the diagnostic potentials of the other three recombinant proteins were less effective compared to rSJCHGC05668.

### Development and application of rSJCHGC05668-indirect ELISA for diagnosis of *S. japonicum*-infected/uninfected human sera

**Figure 5:**
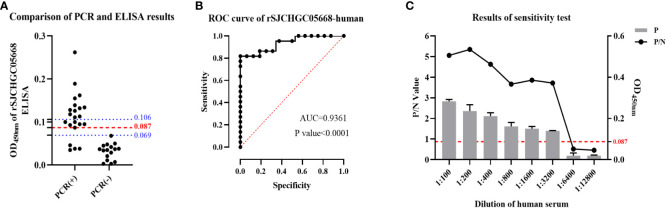
Development and application of rSJCHGC05668-indirect ELISA for diagnosis of human (n = 38) infected/uninfected with *S. japonicum* sera and comparison with PCR results using cfDNA extracted from these human sera as templates. **(A)** Comparison of PCR results using cfDNA extracted from human sera as templates and rSJCHGC05668-indirect ELISA results. **(B)** The ROC curve of rSJCHGC05668-indirect ELISA results. **(C)** The results of the *S. japonicum*-infected human sera sensitivity test. Data are represented as means ± S.E.

According to the results of the above detection of mice sera, we established rSJCHGC05668-indirect ELISA for the diagnosis of *S. japonicum*-infected/uninfected human sera. ELISA conditions were optimized and the best conditions (coating concentration 4 μg/ml, serum dilution 1:200, secondary antibody dilution 1: 5000 and reaction time of substrate solution 15 min) were subsequently used (**Supplementary Figures 1A-C**). Using established rSJCHGC05668-indirect ELISA to detect the 16 human serum samples which were identified as negative for *S. japonicum*-infection by PCR using cfDNA extracted from human sera as templates (**Figure 5A**). The average and standard deviation (SD) of absorbance at 450 nm was calculated, the cut-off value was considered as $\overset{–}{x}+3SD$ . Besides, to avoid false positive results, a suspicious interval ( $\overset{–}{x}+2SD$ , $\overset{–}{x}+4SD$ ) was set. Compared with PCR results, 16 samples identified as negative for *S. japonicum*-infection by PCR were also detected as negative by ELISA, while among 22 samples identified as positive for *S. japonicum*-infection by PCR, 4 samples were detected as negative, 6 samples were detected as suspected positive and 12 samples were detected as positive by ELISA. The diagnostic potential of the rSJCHGC05668 recognized by human serum was preliminarily evaluated by Receiver Operating Characteristic (ROC) analysis (**Figure 5B**). The Area under Curve (AUC) indicated its excellent diagnostic capability. The positive and negative sera were ratio diluted to test the sensitivity of the human sera for this method was 1:3200 (**Figure 5C**). There are 5 human serum samples were selected randomly to detect and evaluate the repeatability of intra and inter-batch test results (**Table 1**). The intra and inter-batch coefficient of variation (CV) of the established rSJCHGC05668-indirect ELISA was less than 15%, with good repeatability and stable detection results.

**Table 1:** Repeatability test of rSJCHGC05668-indirect ELISA.

Sample No.	Intra batch CV%		Inter batch CV%	
	$\bar{\text{x}}$ ±s	CV	$\bar{\text{x}}$ ±s	CV
1	0.156±0.002	1.11	0.150±0.008	5.29
2	0.091±0.002	1.68	0.089±0.002	1.95
3	0.118±0.001	0.98	0.111±0.013	11.42
4	0.092±0.002	1.88	0.090±0.006	6.14
5	0.102±0.003	2.59	0.099±0.010	9.98

## Discussion

Significant progress has been achieved in controlling schistosomiasis japonica in China, but it remains an important public health concern (Gray et al., 2010). Current standard diagnostic tool for schistosomiasis still depends on stool microscopy such as the Kato-Katz method recommended by the World Health Organization (World Health Organization, 2002). However, it is difficult to detect the infection in low-endemic areas using the traditional parasitological method (Wang et al., 2006). The development of new diagnostic tools is important to prevent and control this disease. Recent studies have focused on serological methods to detect *S. japonicum* infection. To minimize the cross-reactions with other parasites and hosts, searching for effective *S. japonicum*-specific proteins as diagnostic antigens have attracted recent attentions and several recombinant proteins have been tested for their diagnostic potential (Sangfuang et al., 2016; Wang et al., 2018).

*S. japonicum* EV has been extensively studied in recent years for its regulatory role in host-parasite interaction (Zhu et al., 2016). Recent studies have suggested that *S. mansoni* EVs and its cargo have the potential for diagnosis of schistosomiasis (Sotillo et al., 2016; Samoil et al., 2018). We have previously identified several proteins isolated from *S. japonicum* EVs (Zhu et al., 2016) and demonstrated that *Sj*EV proteins could serve as potential diagnostic markers (Chen et al., 2020). However, our knowledge regarding the role of *Sj*EV proteins is very limited. This study aims to investigate four *S. japonicum* EV proteins and evaluate their potential for diagnosing schistosomiasis. SJHYP, SJCHGC02838, SJCHGC05593 and SJCHGC05668 were selected because they all possess signal peptides and were predicted to be *Sj*EVs structure-associated and secreted proteins (Zhu et al., 2016), one of which has been demonstrated to have diagnostic potential previously (Wang et al., 2018).

In this study, we firstly investigated transcript levels of these four proteins at different stages of *S. japonicum* (**Figure 2**). qRT-PCR results showed that the transcript profiles of these four *Sj*EV proteins shared a similar expression pattern with the known *Sj*EV protein CD63, which exhibited expression across the developmental stages of *S. japonicum*, with higher expression levels in adult stages compared to that in eggs and cercariae. In a previous study, it was shown that *Sj*EV protein CD63 can be specifically recognized by serum samples from *S. japonicum*-infected mice (Wang et al., 2018). To investigate whether these four proteins could be recognized by *S. japonicum*-infected serum samples, we produced four recombinant proteins and investigated their interaction with serum samples collected at different time points post-infection. Western blot and ELISA results showed that all four recombinant proteins could be specifically recognized by serum samples from *S. japonicum*-infected mice as positive at 22 and 28 dpi and rSJCHGC05668 could be diagnosed as positive as early as 5 dpi (**Figure 3**). The diagnostic potentials of the four *Sj*EV proteins varied, the mixtures were highest but the rSJCHGC05668 showed very close results (**Figure 4**).

We developed and applied the rSJCHGC05668-indirect ELISA for the diagnosis of clinical serum samples of human infected/uninfected with *S. japonicum* and compared them with PCR results using cfDNA extracted from these human sera as templates (**Figure 5**). There were some differences in the detection results of the two experimental methods for the same human serum samples probably due to separate techniques for different target molecules. Although compared with cfDNA methods, the rSJCHGC05668-indirect ELISA showed a low detectable rate, ELISA is used as a preferential method due to its low-cost.

These results further confirmed that the *Sj*EV proteins at least those four selected proteins including SJHYP, SJCHGC02838, SJCHGC05593 and SJCHGC05668 have potential for diagnosing schistosomiasis. Especially, it was shown that rSJCHGC05668 protein served as a good biomarker for early diagnosis, even at low levels of infection.

## Conclusions

In the present study, we screened the previously identified *S. japonicum* EV proteins and selected 4 proteins to evaluate their potential for schistosomiasis diagnosis. The results of Western blot and ELISA showed detectable antibody levels against the four selected antigens in mice infected with *S. japonicum*. In addition, we developed and applied the rSJCHGC05668-indirect ELISA for the diagnosis of human clinical serum samples infected/uninfected with *S. japonicum* and compared with PCR results using cfDNA extracted from same samples. Our results indicated that *S. japonicum* EV proteins could be potentially effective biomarkers for diagnosing schistosomiasis.

## Data availability statement

The original contributions presented in the study are included in the article/**Supplementary Material**. Further inquiries can be directed to the corresponding author.

## Ethics statement

The studies involving humans were approved by Shanghai Tenth People’s Hospital, Tongji University. The studies were conducted in accordance with the local legislation and institutional requirements. The human samples used in this study were acquired from gifted from another research group. Written informed consent for participation was not required from the participants or the participants’ legal guardians/next of kin in accordance with the national legislation and institutional requirements. The animal study was approved by Shanghai Veterinary Research Institute, Chinese Academy of Agricultural Sciences. The study was conducted in accordance with the local legislation and institutional requirements.

## Author contributions

HW: Data curation, Formal Analysis, Investigation, Methodology, Software, Validation, Writing – original draft. BG: Data curation, Formal Analysis, Writing – review & editing. HL: Data curation, Writing – review & editing, Investigation, Methodology, Resources. YZ: Resources, Writing – review & editing. XY: Resources, Writing – review & editing. GC: Resources, Writing – review & editing, Conceptualization, Funding acquisition, Project administration.

## Supplementary material

Supplementary Figure 1 Development of rSJCHGC05668-indirect ELISA for diagnosis of *S. japonicum* infected/uninfected human sera. **(A)** Exploration of rSJCHGC05668 coating concentration (2, 4 and 6 μg/mL) and human serum dilution (1:100/200/400/800). **(B)** Determination of the optimal dilution of the secondary antibody (1:2500/5000/10000/20000). **(C)** Determination of the optimal reaction time (5, 10, 15 and 20 min) of substrate solution.
