# Supplementary figures and images for: Schistosoma japonicum extracellular vesicle proteins serve as effective biomarkers for diagnosing parasite infection
Source: Front Cell Infect Microbiol. 2024 May 16;14:1391168. doi: 10.3389/fcimb.2024.1391168 (PMC11137203; doi:10.3389/fcimb.2024.1391168)

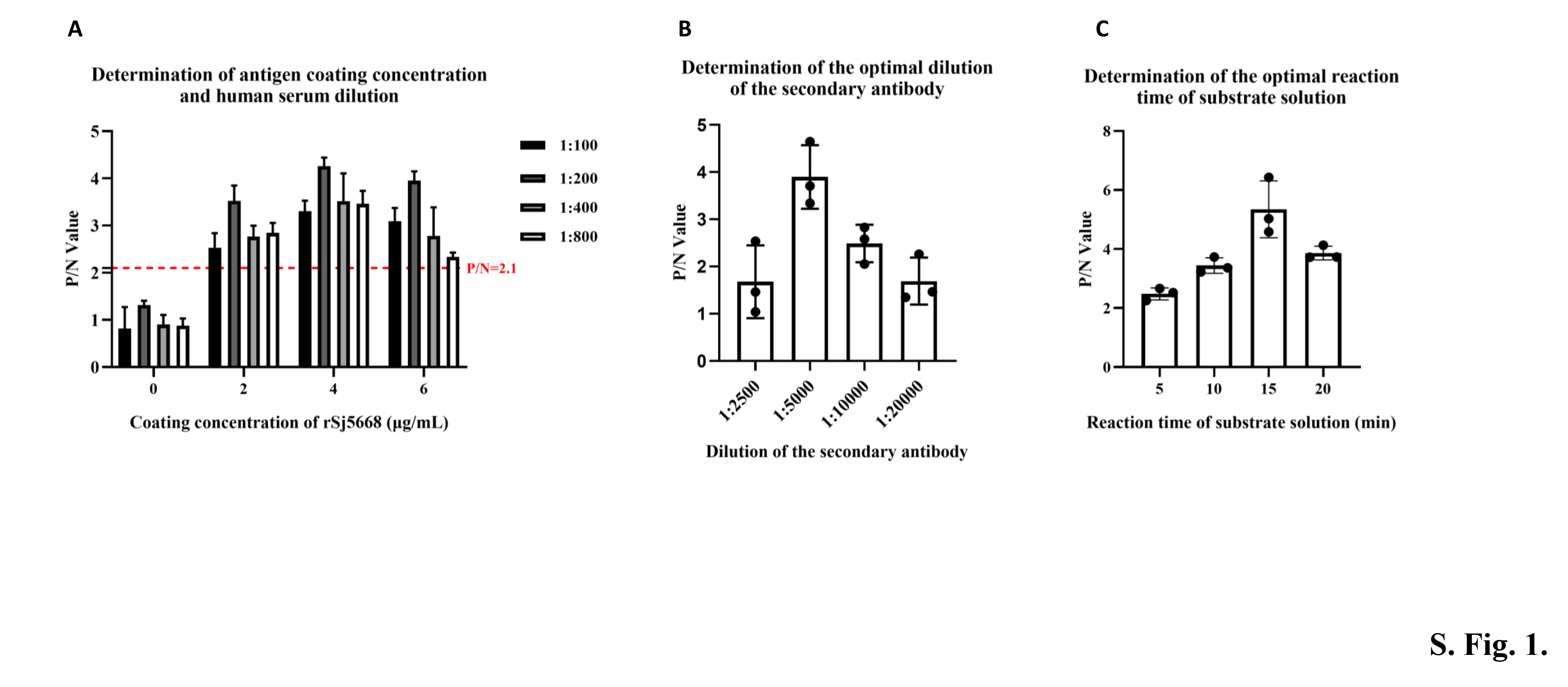

Supplement: Supplementary Figure 1 — Development of rSJCHGC05668-indirect ELISA for diagnosis of S. japonicum infected/uninfected human sera. (A) Exploration of rSJCHGC05668 coating concentration (2, 4 and 6 μg/mL) and human serum dilution (1:100/200/400/800). (B) Determination of the optimal dilution of the secondary antibody (1:2500/5000/10000/20000). (C) Determination of the optimal reaction time (5, 10, 15 and 20 min) of substrate solution. [file Image_1.tif]
